# Glycyrrhizic Acid-Modified Gold Nanoparticles Show Inhibitory Activity Against PRRSV and SARS-CoV-2 Pseudovirus In Vitro

**DOI:** 10.3390/v18040454

**Published:** 2026-04-09

**Authors:** Ting Tong, Xiaotong Zhang, Yating Lei, Linjie Li, Shaobo Xiao, Jiangong Liang

**Affiliations:** 1 School of Life and Health Sciences, Hunan University of Science and Technology, Xiangtan 411201, China; 2 College of Chemistry, Huazhong Agricultural University, Wuhan 430070, China; 3 National Key Laboratory of Agricultural Microbiology, College of Veterinary Medicine, Huazhong Agricultural University, Wuhan 430070, China

**Keywords:** gold nanoparticles, glycyrrhizic acid, antiviral, PRRSV, SARS-CoV-2

## Abstract

The development of novel antiviral nanomaterials is an important approach for addressing emerging viral threats. In this study, glycyrrhizic acid-modified gold nanoparticles (GA-Au NPs) were successfully synthesized and characterized, and their inhibitory effects against porcine reproductive and respiratory syndrome virus (PRRSV) and severe acute respiratory syndrome coronavirus 2 (SARS-CoV-2) pseudovirus were systematically evaluated. At non-cytotoxic concentrations, GA-Au NPs showed inhibitory activity against PRRSV in vitro. Stage-specific assays suggested that intracellular replication-related events were prominently affected, with additional inhibitory effects observed during adsorption, invasion, and release, whereas no direct virucidal activity was detected under the tested conditions. Furthermore, GA-Au NPs dose-dependently reduced SARS-CoV-2 pseudovirus infection-associated reporter signals in HEK-293T-ACE2 cells, supporting inhibitory activity in an additional viral model. In conclusion, GA-Au NPs represent a biocompatible antiviral nanomaterial with multi-stage inhibitory activity against PRRSV and inhibitory effects in a SARS-CoV-2 pseudovirus model, supporting their further evaluation as antiviral nanomaterials in enveloped virus-related models.

## 1. Introduction

Viral diseases remain major threats to both public health and animal production, causing substantial social and economic burdens worldwide [1]. The continuing emergence and re-emergence of pathogenic viruses, exemplified by porcine reproductive and respiratory syndrome virus (PRRSV) in swine and severe acute respiratory syndrome coronavirus 2 (SARS-CoV-2) in humans, underscore the need for antiviral strategies that remain effective in the face of viral variation and immune evasion [2,3]. Although vaccination is indispensable for viral disease control, its protective efficacy may be compromised by rapid viral evolution, incomplete cross-protection, or the limited availability of broadly effective vaccine formulations [4,5]. These challenges have prompted increasing interest in complementary antiviral approaches that can directly interfere with the viral infection process.

PRRSV is one of the most economically important viral pathogens in the global swine industry. This enveloped, positive-sense single-stranded RNA virus causes reproductive failure in sows and respiratory disorders in piglets and growing pigs, resulting in persistent and often difficult-to-control outbreaks [6]. The control of PRRSV remains particularly challenging because of its high genetic variability, frequent immune evasion, and pronounced tropism for porcine alveolar macrophages, which facilitates viral persistence and undermines host antiviral defenses [7,8]. Despite continued efforts in vaccine development, the protection conferred by existing vaccines against heterologous strains is often suboptimal, and effective antiviral therapeutics for PRRSV are still lacking [9,10]. In parallel, the COVID-19 pandemic has further highlighted the vulnerability of human populations to rapidly evolving enveloped RNA viruses [11,12,13]. Together, these concerns provide a strong rationale for exploring antiviral platforms with mechanisms distinct from conventional vaccine-based protection.

Nanomaterials have emerged as promising tools for antiviral research because their physicochemical properties can be tailored to influence virus–host interactions and to improve the delivery of bioactive agents [14,15,16,17]. Among them, gold nanoparticles (Au NPs) are particularly attractive owing to their controllable size, facile surface functionalization, and generally favorable biocompatibility [18,19]. Au NPs exert antiviral activity through versatile mechanisms. They can directly interact with viral surfaces or host cell receptors, competitively inhibiting viral attachment and cellular entry [20,21]. Au NPs have been investigated in antiviral applications as inhibitors of viral attachment or entry and as carriers for antiviral cargos, including drugs, nucleic acids, and peptides [22,23,24]. However, unmodified Au NPs often show limited biological specificity, and their intrinsic antiviral activity is usually insufficient for efficient virus inhibition [25]. Surface engineering with biologically active ligands is therefore an important strategy to enhance their antiviral performance and expand their functional utility [26].

Glycyrrhizic acid (GA), a natural triterpenoid saponin derived from licorice root, exhibits diverse pharmacological properties, including anti-inflammatory, immunomodulatory, and broad-spectrum antiviral activities [27,28], which have been attributed to its ability to modulate membrane fluidity and inhibit viral entry [29,30,31]. Previous studies have shown that GA can inhibit multiple viruses, including HIV, influenza virus, and PRRSV, and its antiviral effects have been linked to interference with membrane-associated events and viral entry-related processes [32,33,34]. In view of its established bioactivity and natural origin, GA is an appealing candidate for integration into nanomaterial-based antiviral systems. Nevertheless, while GA-derived carbon-based nanomaterials have shown antiviral potential [35], the development of GA-functionalized metal nanoparticles for antiviral applications remains insufficiently explored.

In this study, we synthesized glycyrrhizic acid-modified gold nanoparticles (GA-Au NPs) and characterized their physicochemical properties. We then evaluated their antiviral activity in vitro using PRRSV as the primary model and further examined their inhibitory effect in a SARS-CoV-2 pseudovirus entry system. By combining nanomaterial design with virological evaluation across multiple infection-related stages, this work aimed to assess the potential of GA-Au NPs as an antiviral nanoplatform and to provide experimental evidence for their further development against enveloped viral infections.

## 2. Materials and Methods

### 2.1. Synthesis and Purification of GA-Au NPs

GA-Au NPs were synthesized following a previously reported method with modifications [36]. Briefly, 10.0 mL of an aqueous glycyrrhizic acid solution (1.0 mg/mL) and 10.0 mL of an HAuCl_4_·3H_2_O solution (1.0 mmol/L) were prepared separately. The HAuCl_4_ solution was heated in a sand bath to a specific temperature (a series of temperatures was tested for optimization). Upon reaching the target temperature, the GA solution was rapidly added under vigorous stirring. At 10 min, the pH was adjusted to 8.0 with NaOH (0.1 mol/L), triggering an immediate color change. Following further reaction at room temperature under stirring for the prescribed time, the crude product was dialyzed (MWCO: 3.5 kDa) against deionized water for 24 h (with water refreshed every 8 h) to eliminate unreacted precursors and low-MW impurities. The purified GA-Au NPs were then passed through a 0.22 μm filter (SLGP033R, Millipore, Burlington, MA, USA) for sterilization prior to cell-based experiments and stored at 4 °C.

### 2.2. Cells and Viruses

MARC-145 cells, a subclone of the African green monkey kidney cell line MA-104, have been widely used in PRRSV research. In our study, the MARC-145 cells were obtained from a well-established stock maintained in our laboratory, which was originally acquired from a commercial source. PRRSV strain WUH3 (GenBank accession no. HM853673), a highly pathogenic type 2 (North American) PRRSV, was previously isolated from the brains of pigs suffering from a high fever syndrome in China at the end of 2006 [37]. HEK-293T cells, HEK-293T-ACE2 cells, and the SARS-CoV-2 pseudovirus (Catalog Number: FNV215) were purchased from Fubio Biomedical Technology Co., Ltd. (Suzhou, China).

MARC-145 cells, HEK-293T cells, and HEK-293T-ACE2 cells (ACE2-expressing 293T cells) were cultured in DMEM/HIGH GLUCOSE (HyClone) containing 10% fetal bovine serum (FBS, PAN) at 37 °C with 5% CO_2_ in a humidified incubator. PRRSV was amplified, and the titer was determined in MARC-145 cells by plaque reduction assay.

### 2.3. Cytotoxicity Assay (MTT Assay)

The cytotoxicity of GA-Au NPs was evaluated on MARC-145 cells using the standard MTT assay. Briefly, cells were seeded in 96-well plates and grown to 80–90% confluency. The culture medium was replaced with fresh medium containing various concentrations of GA-Au NPs (0, 50, 100, 150, 300 µg/mL), while cells treated with DMEM supplemented with 2% fetal bovine serum (FBS) served as the negative control. After incubation for 12, 24, 36, and 48 h, the medium was aspirated, and each well was replenished with 100 µL of fresh DMEM (2% FBS) and 20 µL of MTT solution (5 mg/mL). Following a 4 h incubation at 37 °C, the resulting formazan crystals were dissolved by adding 150 µL of dimethyl sulfoxide (DMSO) per well. The optical density (OD) of each well was measured at 630 nm using a microplate reader (SpectraMax i3x, Molecular Devices, San Jose, CA, USA).

### 2.4. Antiviral Activity Assay Against PRRSV

The antiviral efficacy of GA-Au NPs against PRRSV was evaluated as follows: MARC-145 cells were pre-treated with varying concentrations of GA-Au NPs (0, 50, 100, and 150 µg/mL) for 2 h at 37 °C. Concurrently, PRRSV was pre-incubated with identical concentrations of GA-Au NPs for 1 h at 4 °C. Cells were then infected with the pre-treated virus at a multiplicity of infection (MOI) of 1.0 for 1 h at 37 °C. Following removal of the inoculum and three washes with PBS, cells were maintained in medium containing the corresponding concentration of GA-Au NPs.

For qualitative assessment, the antiviral effect was evaluated at 12, 24, 36, and 48 h post-infection (hpi) via indirect immunofluorescence assay (IFA) for the detection of the viral N protein.

For quantitative assessment, samples were collected at the same time points. The culture supernatant was harvested and replaced with an equal volume of fresh medium. Cells were subjected to three cycles of freeze–thaw to prepare the cell lysate. Viral titers in both the supernatant and the cell lysate were determined by plaque assay.

### 2.5. Direct Inactivation Assay

A direct viral inactivation assay was performed to evaluate whether GA-Au NPs could inactivate free PRRSV particles. Briefly, the virus (MOI = 0.1) was mixed with GA-Au NPs (0, 50, 100, 150 µg/mL) and incubated for 1 h at 37 °C. Following incubation, the mixture was added to cells for a 1 h infection period. The inoculum was then discarded, and the monolayer was washed three times with PBS; the plaque assay was then performed to determine the viral titer.

### 2.6. Viral Adsorption Assay

The effect of GA-Au NPs on the PRRSV adsorption stage was evaluated. Briefly, confluent monolayers of MARC-145 cells in 6-well plates were cooled and maintained at 4 °C for 30 min. The cells were then inoculated with a mixture of PRRSV (MOI = 0.1) and different concentrations of GA-Au NPs, followed by incubation for 2 h at 4 °C. This temperature permits viral attachment while blocking internalization. After incubation, the inoculum was removed, and the cells were washed twice to eliminate unbound virus and GA-Au NPs. A plaque assay was then performed to determine the viral titer.

### 2.7. Viral Invasion Assay

MARC-145 cells grown to confluence in 6-well plates were cooled to and maintained at 4 °C for 30 min. Then, the cells were incubated with PRRSV (MOI = 0.1) for 2 h at 4 °C for viral adsorption. Subsequently, after washing with cold PBS to remove unbound virus, fresh medium containing the corresponding concentrations of GA-Au NPs was added. Finally, the temperature was shifted to 37 °C for 3 h to permit viral internalization. The medium was then removed, and the cells were washed twice with ice-cold PBS to eliminate unbound virus and GA-Au NPs. A plaque assay was then performed to determine the viral titer.

### 2.8. Viral Replication Assay

The effect of GA-Au NPs on viral RNA replication was assessed by quantifying negative-strand RNA. Following infection of MARC-145 cells with PRRSV (MOI = 1.0) for 1 h at 37 °C, the inoculum was removed, and the cells were cultured for an additional 6 h. GA-Au NPs were then added at specified concentrations. Cells were further harvested at 7, 8, 9, and 10 h post-treatment. At each time point, total RNA was extracted using Trizol reagent. The level of PRRSV negative-strand RNA was subsequently quantified by reverse transcription quantitative polymerase chain reaction (RT-qPCR). The detailed protocol, including primer sequences (Table S1), is provided in the Supplementary Materials.

### 2.9. Viral Release Assay

MARC-145 cells were inoculated with PRRSV (MOI = 1.0) for 1 h. After removal of the inoculum and washing, cells were cultured for an additional 18 h to allow completion of viral replication. The medium was then replaced with fresh medium containing GA-Au NPs. At 15, 30, 45, and 60 min post-treatment, the culture supernatant (containing released virus) was collected and replaced with an equal volume of fresh medium. At a later time, cells were subjected to three cycles of freeze–thaw to prepare the cell lysate. Viral titers in both the supernatant and the cell lysate were determined by plaque assay.

### 2.10. Antiviral Assay Against SARS-CoV-2 Pseudovirus

The broad-spectrum antiviral activity was tested using a SARS-CoV-2 pseudovirus system. HEK-293T-ACE2 cells were seeded in 24-well plates and pre-treated with various concentrations of GA-Au NPs (0–200 µg/mL) in DMEM (2% FBS) for 2 h. The medium was then replaced with a suspension of SARS-CoV-2 pseudovirus at an MOI of 5.0. After 8 h of infection, the supernatant was discarded, and the cells were maintained in fresh medium for 48 h. The infection efficiency was quantified by measuring the fluorescence intensity of the encoded green fluorescent protein (GFP) using an inverted fluorescence microscope (model IX70; Olympus Corporation, Tokyo, Japan) and by determining the luciferase activity using a Dual-Luciferase^®^ Reporter Assay System, following the manufacturer’s protocol.

### 2.11. Statistical Analysis

Data are presented as the mean of at least three independent biological replicates. In the figures, statistically significant differences between groups are denoted by asterisks: * *p* < 0.05, ** *p* < 0.005, and *** *p* < 0.001. Non-significant differences (*p* > 0.05) are marked as “ns”. Statistical analysis was conducted using one-way ANOVA in Excel (Microsoft Office Home and Student 2019) software, with a significance level (α) set at 0.05.

## 3. Results

### 3.1. Synthesis Optimization and Structural Characterization of GA-Au NPs

To obtain well-dispersed glycyrrhizic acid-functionalized gold nanoparticles (GA-Au NPs), the nanoparticles were synthesized using a modified version of a previously reported method [36], and the reaction conditions were subsequently optimized by varying the heating temperature of the HAuCl_4_ solution and the reaction time after glycyrrhizic acid (GA) addition. UV-Vis spectroscopy was used for the initial screening because the surface plasmon resonance profile is sensitive to nanoparticle size distribution and colloidal uniformity. As summarized in Table S2 and shown in Figure S1, different reaction conditions produced nanoparticles with distinct optical properties and hydrodynamic diameters.

Under condition a, in which the HAuCl_4_ solution was heated to 100 °C, GA was added after 20 min, and the reaction was then continued at 25 °C for 90 min, the resulting suspension displayed a blood-red appearance, a narrow and symmetric absorption peak, and the smallest hydrodynamic diameter (approximately 18.9 nm). By contrast, conditions b–d yielded broader absorption bands and larger particle sizes (25.4–32.8 nm), indicating lower particle uniformity. TEM observations were consistent with these results (Figure S2), showing that nanoparticles prepared under condition a were more spherical and more uniformly dispersed than those obtained under the other conditions. On this basis, 100 °C was selected for further optimization.

The reaction temperature was then fixed at 100 °C, and the total reaction time was varied from 1.5 to 3 h. As shown by the UV-Vis spectra (Figure S3) and TEM images (Figure S4), a total reaction time of 1.5 h produced nanoparticles with the most favorable characteristics, including a sharp absorption peak and high monodispersity. Extending the reaction time did not result in a clear improvement in particle uniformity. Therefore, the optimized synthesis conditions were defined as 100 °C with a total reaction time of 1.5 h, including the 90 min reaction period at 25 °C after GA addition. This protocol was used for the preparation of GA-Au NPs in all subsequent experiments.

**Figure 1:**
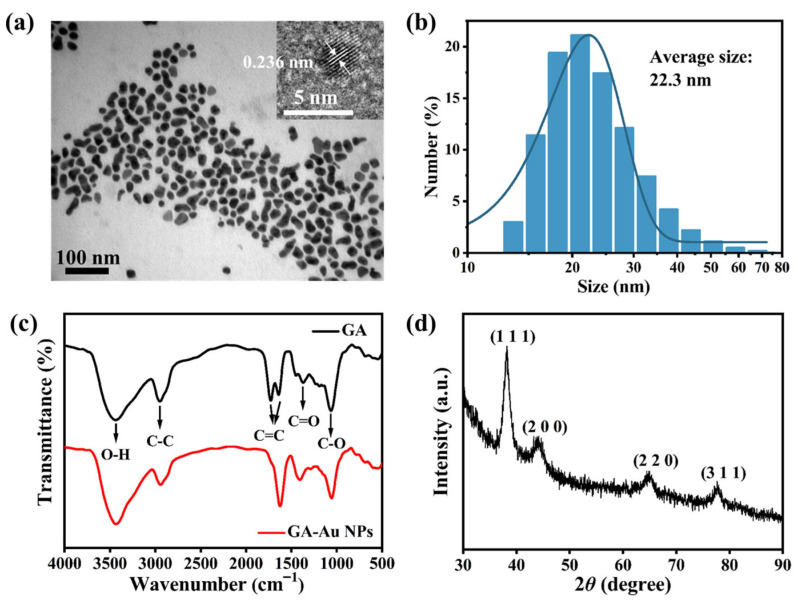
Structural characterization of GA-Au NPs: (**a**) TEM image of GA-Au NPs (inset: HRTEM image showing lattice fringes). (**b**) DLS hydrodynamic diameter distribution of GA-Au NPs. (**c**) FTIR spectra of free GA and GA-Au NPs. (**d**) XRD pattern of GA-Au NPs.

The optimized GA-Au NPs were further characterized to examine their morphology, crystalline structure, and surface chemistry. TEM analysis showed that the nanoparticles were quasi-spherical and well dispersed (Figure 1a). The high-resolution image revealed clear lattice fringes with an interplanar spacing of 0.236 nm, which corresponds to the (111) plane of face-centered cubic gold, indicating the crystalline nature of the Au core [37]. XRD analysis provided further structural support (Figure 1d). The diffraction peaks observed at 38.2°, 44.2°, 64.8°, and 77.6° were assigned to the (111), (200), (220), and (311) planes of face-centered cubic gold, respectively [38], and no obvious impurity peaks were detected. FTIR spectroscopy was used to assess whether GA-derived functional groups were present on the nanoparticle surface (Figure 1c). Pure GA showed characteristic absorption bands at approximately 3432 cm^−1^ (O–H stretching), 2942 cm^−1^ (C–H stretching), 1645 cm^−1^ (C=O-related vibration), 1376 cm^−1^, and 1054 cm^−1^ (C–O-related vibrations). These major bands were retained in the spectrum of GA-Au NPs, supporting the presence of GA-derived functional groups on the nanoparticles [36]. In addition, slight shifts and changes in band shape or intensity were observed, suggesting an interaction between GA and the Au surface rather than simple physical mixing. DLS measurements showed a monomodal size distribution with an average hydrodynamic diameter of approximately 22.3 nm (Figure 1b), which was larger than the particle core size observed by TEM, consistent with the presence of a surface coating and associated hydration shell.

**Figure 2:**
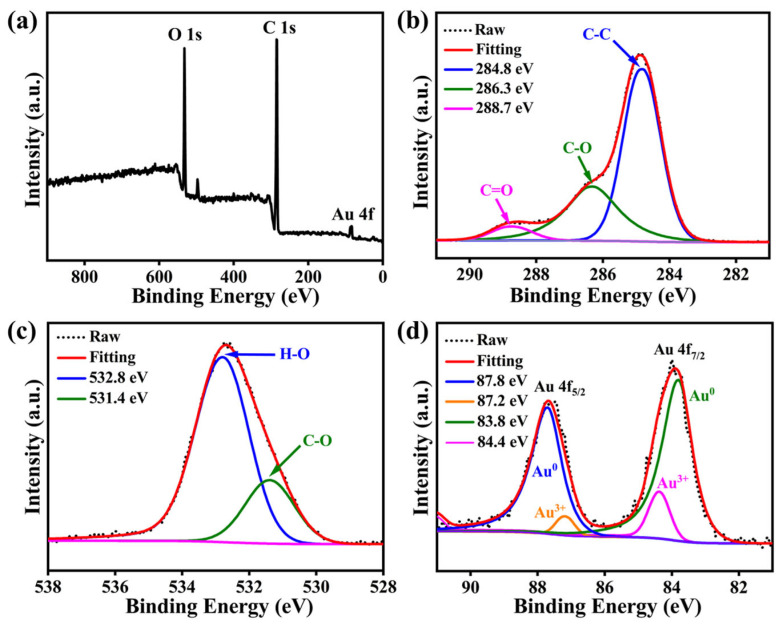
X-ray photoelectron spectroscopy analysis of GA-Au NPs: (**a**) Survey spectrum. (**b**) High-resolution C 1s spectrum. (**c**) High-resolution O 1s spectrum. (**d**) High-resolution Au 4f spectrum.

XPS analysis further supported the surface functionalization of the nanoparticles by GA (Figure 2). The survey spectrum showed the expected Au, C, and O signals, confirming the elemental composition of the nanomaterial (Figure 2a). In the high-resolution C 1s spectrum, the components centered at approximately 284.8, 286.3, and 288.5 eV were assigned to C–C/C–H (~284.8 eV), C–O (~286.3 eV), and C=O/O–C=O (~288.5 eV) species, respectively (Figure 2b). The O 1s spectrum showed corresponding peaks attributable to C=O (~531.5 eV) and C–O/H–O (~532.8 eV) species (Figure 2c), consistent with the presence of oxygen-containing functional groups derived from GA. The Au 4f spectrum was dominated by the doublet of metallic Au^0^, with Au 4f_7/2_ centered at approximately 84.0 eV, indicating an effective reduction of the gold precursor (Figure 2d) [39]. A minor Au^3+^ (Au 4f7/2 at ~85.5 eV) component was also detected. The coexistence of the characteristic carboxylate signal in the C 1s/O 1s spectra and the slight oxidative shift in the Au 4f spectrum suggests a chemical interaction, likely through coordination bonding between the carboxylate groups of GA and the surface of the gold nanoparticles. Collectively, the XPS results are consistent with the presence of GA-derived surface species on the nanoparticles.

**Figure 3:**
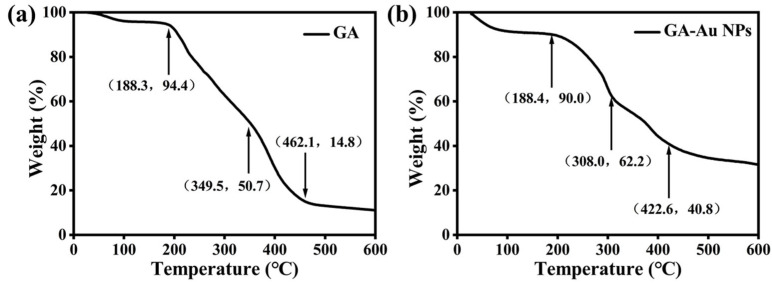
Thermogravimetric analysis of GA (**a**) and GA-Au NPs (**b**).

Thermogravimetric analysis (TGA) was performed to further examine the composition of the nanomaterial (Figure 3). As shown in Figure 3a, the TGA curve of pure glycyrrhizic acid (GA) showed an initial mass loss of approximately 5.6% up to 188.3 °C, attributed to the removal of physically adsorbed water or solvent. Its major thermal decomposition occurred between 200 and 400 °C, with 50.7% residue remaining at 349.5 °C, corresponding to the breakdown of the molecular backbone. The final residue of pure GA stabilized at about 14.8% beyond 462.1 °C, representing its ash content. In contrast, as shown in Figure 3b, the GA-Au NPs composite exhibited a slightly higher initial mass loss (~10% up to 188.4 °C), possibly due to its larger surface area. Notably, its main decomposition step was observed earlier, with 62.2% residue remaining at 308.0 °C, suggesting a decrease in the thermal stability of GA when bound to the Au NP surface, likely due to altered chemical interactions. Most significantly, the final residue of the GA-Au NPs composite stabilized at 40.8% at 422.6 °C, which is substantially higher than that of pure GA. This marked increase is direct evidence for the presence of the thermally stable gold core. Assuming the final residue consists primarily of elemental gold, the Au mass fraction is estimated to be approximately 40.8%, allowing for the calculation of the GA loading on the nanoparticles.

Overall, the UV-Vis, TEM, DLS, FTIR, XRD, XPS, and TGA results collectively support the successful preparation of well-dispersed GA-Au NPs with defined crystallinity, surface-associated GA-derived chemical features, and measurable GA loading.

### 3.2. Cytotoxicity Assay of GA-Au NPs

**Figure 4:**
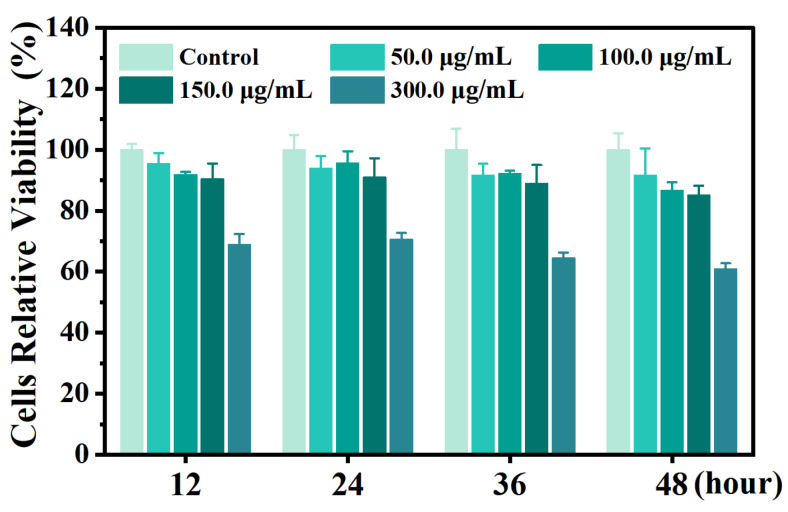
Cytotoxicity of GA-Au NPs at different concentrations and times as detected by MTT assay. Error bars represent the standard deviation of three independent replicates.

Cytocompatibility is an essential prerequisite for evaluating the antiviral potential of a candidate nanomaterial. To assess the effects of GA-Au NPs on host cells, MARC-145 cell viability was measured by MTT assay after exposure to 0, 50, 100, 150, and 300 μg/mL GA-Au NPs for 12, 24, 36, and 48 h. As shown in Figure 4, cell viability exhibited concentration- and time-dependent changes following treatment with GA-Au NPs. Within the concentration range of 50–150 μg/mL, MARC-145 cell viability remained above 85% at all tested time points, indicating acceptable cytocompatibility under the present experimental conditions. In contrast, treatment with 300 μg/mL for 48 h reduced cell viability to below 70%, suggesting increased cytotoxicity at the highest concentration and longest exposure time examined.

Based on these results, 150 μg/mL was considered the upper concentration limit for subsequent antiviral assays in MARC-145 cells, and 50, 100, and 150 μg/mL were selected for further study. This concentration range allowed the antiviral effects of GA-Au NPs to be evaluated under conditions with limited interference from overt cytotoxicity.

### 3.3. Antiviral Activity of GA-Au NPs Against PRRSV Infection

**Figure 5:**
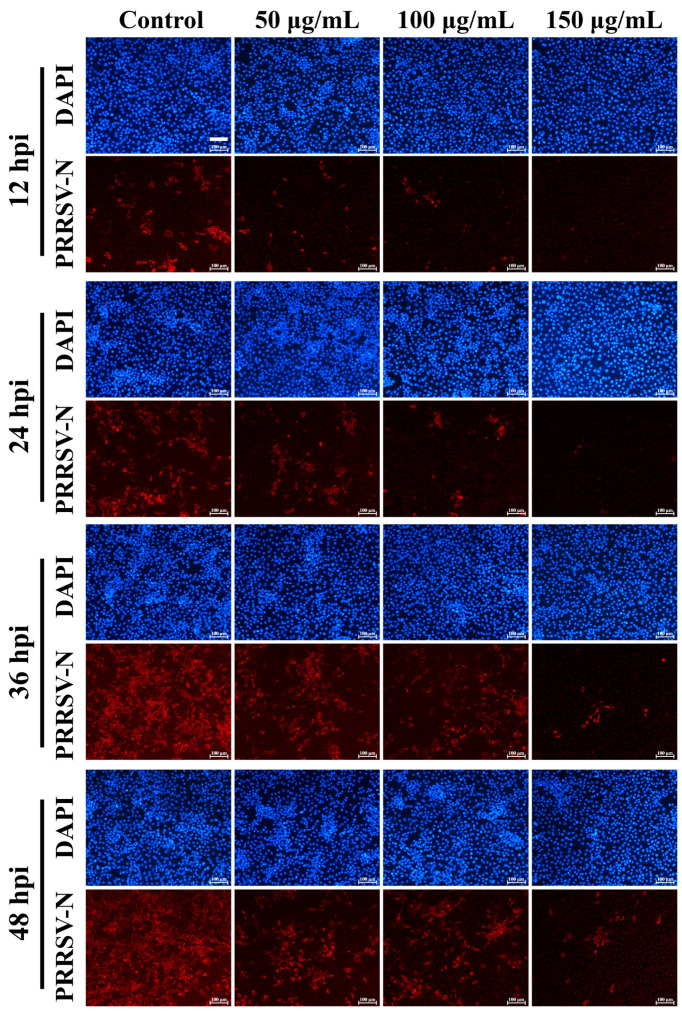
Indirect immunofluorescence analysis of the inhibitory effects of GA-Au NPs on PRRSV infection in MARC-145 cells. MARC-145 cells were infected with PRRSV (MOI = 1.0) for 1 h, treated with the indicated concentrations of GA-Au NPs, and then cultured for 12, 24, 36, or 48 h. Viral infection was assessed by indirect immunofluorescence staining of the PRRSV N protein (red), and cell nuclei were counterstained with DAPI (blue). In the infected control group without GA-Au NP treatment, the intensity and distribution of red fluorescence increased over time. In contrast, GA-Au NP treatment resulted in a concentration-dependent reduction in N protein fluorescence, with the strongest inhibition observed at 150 μg/mL. Scale bar = 100 μm.

To evaluate the antiviral activity of GA-Au NPs against PRRSV, MARC-145 cells were infected with PRRSV and subsequently treated with different concentrations of GA-Au NPs according to the procedure described in Section 2.4. The inhibitory effect of GA-Au NPs was first examined by indirect immunofluorescence assay (IFA). As shown in Figure 5, the horizontal axis represents the different treatment groups, including the infected control group without GA-Au NP treatment and the groups treated with 50, 100, and 150 μg/mL GA-Au NPs after infection. The vertical axis represents different time points after infection, including 12, 24, 36, and 48 h post-infection (hpi). In each panel, red fluorescence indicates the PRRSV nucleocapsid (N) protein, serving as a marker of viral infection and accumulation in cells, whereas blue fluorescence represents DAPI-stained nuclei and provides a reference for cell localization and density.

The IFA images revealed a clear inhibitory effect of GA-Au NPs on PRRSV infection. In the infected control group, the red fluorescence signal gradually increased from 12 to 48 hpi and became more widely distributed over time, indicating active viral replication and spread in the absence of treatment. By contrast, treatment with GA-Au NPs resulted in a concentration-dependent reduction in both the intensity of the N protein signal and the proportion of infected cells at each observed time point. This inhibitory trend was particularly evident at higher concentrations. In the 150 μg/mL treatment group, the N protein signal was markedly reduced throughout the observation period and remained barely detectable even at 48 hpi, indicating effective suppression of viral protein accumulation under these experimental conditions. In addition, within each treatment group, the inhibitory effect was maintained over time, as the fluorescence signal remained substantially weaker than that observed in the infected control group. Importantly, DAPI staining showed no obvious loss of cell density or apparent nuclear morphological abnormalities across the tested concentrations, suggesting that the antiviral effect observed by IFA was unlikely to result from overt cytotoxicity during the experimental period.

**Figure 6:**
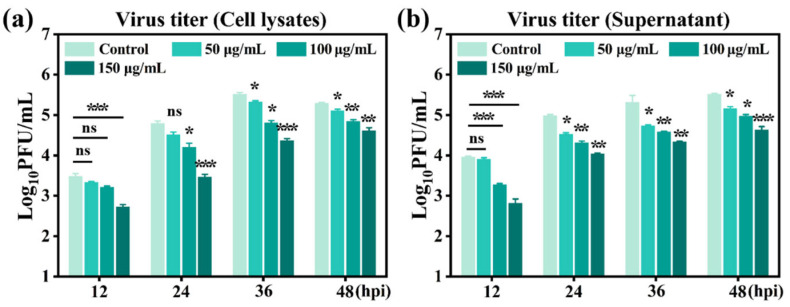
Quantitative analysis of the inhibitory effects of GA-Au NPs on PRRSV production in MARC-145 cells. Infectious PRRSV titers were determined by plaque assay in (**a**) cell culture supernatants and (**b**) cell lysates collected at the indicated time points after infection and treatment with GA-Au NPs. Viral titers in both fractions decreased in a concentration-dependent manner following GA-Au NP treatment. Data are presented as mean ± SD from three independent experiments. *p* values were calculated using one-way ANOVA. * *p* < 0.05, ** *p* < 0.01, *** *p* < 0.001, ns: not significant.

To further quantify the antiviral effect observed, plaque assays were performed to determine infectious PRRSV titers in both cell culture supernatants and cell lysates at different time points after infection. As shown in Figure 6, in the infected control group without GA-Au NP treatment, viral titers in both fractions increased progressively over time, indicating ongoing viral production and release. In contrast, treatment with GA-Au NPs caused a clear concentration-dependent reduction in viral titers at all measured time points. Among the tested concentrations, 150 μg/mL showed the strongest inhibitory effect. Notably, in the 100 and 150 μg/mL groups, the increase in viral titer over the 48 h observation period was markedly attenuated, suggesting sustained suppression of PRRSV replication-associated viral output. The concurrent reduction in viral titers detected in both cell lysates and supernatants indicates that GA-Au NPs reduced the accumulation of intracellular infectious virus as well as the amount of virus released into the culture medium. Under the tested conditions, treatment with 150 μg/mL GA-Au NPs reduced virus infectivity by up to approximately 1.5 log units relative to the infected control.

Taken together, the qualitative observations from IFA and the quantitative plaque assay results consistently demonstrate that GA-Au NPs inhibit PRRSV infection in MARC-145 cells in a concentration-dependent manner.

### 3.4. Stage-Specific Effects of GA-Au NPs on PRRSV Infection

**Figure 7:**
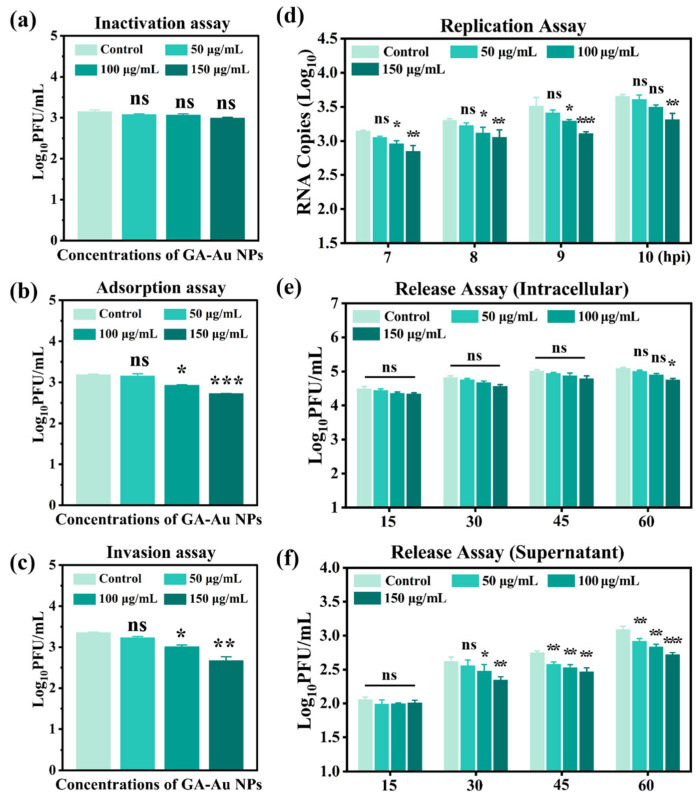
Stage-specific effects of GA-Au NPs on PRRSV infection: (**a**) Direct inactivation assay. PRRSV was pre-incubated with the indicated concentrations of GA-Au NPs for 1 h at 37 °C before infection, and viral infectivity was determined by plaque assay. (**b**) Adsorption assay. MARC-145 cells were incubated with PRRSV and GA-Au NPs at 4 °C for 2 h to allow viral attachment, and cell-associated infectious virus was quantified by plaque assay. (**c**) Internalization assay. After viral adsorption at 4 °C, cells were shifted to 37 °C in the presence of GA-Au NPs to permit viral internalization, and viral infectivity was measured by plaque assay. (**d**) Replication assay. Following viral entry, infected cells were treated with GA-Au NPs, and intracellular PRRSV negative-strand RNA levels were measured by RT-qPCR. (**e**,**f**) Release assay. Infected cells were treated with GA-Au NPs during the late stage of infection, and infectious virus in the culture supernatant (**e**) and cell-associated fraction (**f**) were quantified by plaque assay. Data are presented as mean ± SD from three independent experiments. Statistical significance was determined relative to the untreated control group. *p* values were calculated using one-way ANOVA. * *p* < 0.05, ** *p* < 0.01, *** *p* < 0.001, ns: not significant.

To investigate whether GA-Au NPs directly inactivate PRRSV particles, the virus was pre-incubated with GA-Au NPs for 1 h at 37 °C before inoculation onto cells. As shown in Figure 7a, no significant reduction in viral titer was observed at any tested concentration compared with the untreated virus control group. These results indicate that GA-Au NPs did not exhibit detectable direct virucidal activity against free PRRSV particles under the tested conditions.

We next evaluated whether GA-Au NPs affect viral adsorption. Cells were incubated with a mixture of PRRSV and GA-Au NPs for 2 h at 4 °C, a condition that allows viral attachment but prevents internalization. After removal of unbound virus, a dose-dependent reduction in cell-associated infectious virus was observed relative to the untreated control, with the 150 µg/mL group showing the greatest decrease of approximately 0.5 log (Figure 7b). These findings suggest that GA-Au NPs interfere with PRRSV attachment-related events at the cell surface.

Following synchronized virus adsorption at 4 °C, cells were treated with GA-Au NPs during a subsequent incubation at 37 °C to allow viral internalization. As shown in Figure 7c, treatment with GA-Au NPs resulted in a dose-dependent reduction in the titer of internalized, infectious virus. While the 50 µg/mL concentration did not cause a statistically significant change, treatment with 100 and 150 µg/mL GA-Au NPs led to a significant decrease. The inhibition was most pronounced at 150 µg/mL. This result indicates that, beyond potentially blocking attachment, GA-Au NPs also inhibit a post-attachment step required for successful viral entry into the cell.

To assess whether GA-Au NPs influence viral RNA replication, infected cells were treated with GA-Au NPs after the entry phase, and intracellular viral RNA levels were quantified at the indicated time point. As shown in Figure 7d, GA-Au NP treatment significantly reduced intracellular PRRSV RNA levels in a dose-dependent manner, with the most pronounced inhibition observed at 150 µg/mL. Note that in Figure 7d, the *y*-axis label “Log_10_ RNA copies” corresponds to the amount of intracellular viral negative-strand RNA, determined by RT-qPCR. These results indicate that GA-Au NPs suppress PRRSV infection at a post-entry stage associated with viral RNA replication.

We further evaluated whether GA-Au NPs affect the late stage of viral replication, for which a viral release assay was performed. The infectious virus titers in both the cell lysate (representing the intracellular virus) and the culture supernatant (representing released virus) were quantified at 15, 30, 45, and 60 min after the addition of GA-Au NPs to previously infected cells. As shown in Figure 7e, the titer of cell-associated infectious virus remained relatively constant over the 60-min observation period and showed no statistically significant reduction at any concentration of GA-Au NPs (0, 50, 100, and 150 µg/mL) compared to the virus control. This indicates that GA-Au NPs did not promote the degradation or inactivation of preformed infectious virions inside the cells. In striking contrast, a potent and dose-dependent inhibition of virus release into the supernatant was observed (Figure 7f). Treatment with 150 µg/mL GA-Au NPs resulted in a highly significant reduction in supernatant viral titers at all time points tested. A significant inhibitory effect was also evident at 100 µg/mL, particularly at later time points (45 and 60 min). The distinct outcomes of the release assay for intracellular versus supernatant virus clarify the mechanism of action at this late stage. The lack of effect on the intracellular virus pool demonstrates that GA-Au NPs do not act by clearing pre-assembled virions from within the cell. Instead, the significant suppression of the virus in the supernatant provides direct evidence that GA-Au NPs specifically hinder the process of viral egress.

Collectively, these results indicate that the antiviral activity of GA-Au NPs against PRRSV is mainly associated with interference in viral adsorption and post-entry stages, particularly those related to viral RNA replication and late-stage virus release, rather than direct inactivation activity.

### 3.5. Inhibitory Effect of GA-Au NPs on SARS-CoV-2 Pseudovirus Infection In Vitro

Given the significant antiviral activity of GA-Au NPs against PRRSV, we next examined whether their inhibitory effect could also be observed in an additional viral model relevant to human infection. To this end, a well-established SARS-CoV-2 pseudovirus system was employed to safely evaluate infection-related events in vitro [40]. The SARS-CoV-2 pseudotyped virus was commercially obtained as a ready-to-use reagent. The pseudovirus particles are produced using a lentiviral (HIV-1-derived) packaging system and are pseudotyped with the SARS-CoV-2 spike glycoprotein on their envelope. The replication-incompetent viral genome is based on a transfer plasmid containing a reporter gene expression cassette. This cassette comprises the firefly luciferase and green fluorescent protein genes, which are linked by an internal ribosome entry site (IRES) and whose expression is driven by a cytomegalovirus promoter. Following pseudovirus infection, reverse transcription and genomic integration of the reporter construct enable reporter gene expression, allowing infection efficiency to be evaluated by fluorescence imaging and chemiluminescence readouts.

**Figure 8:**
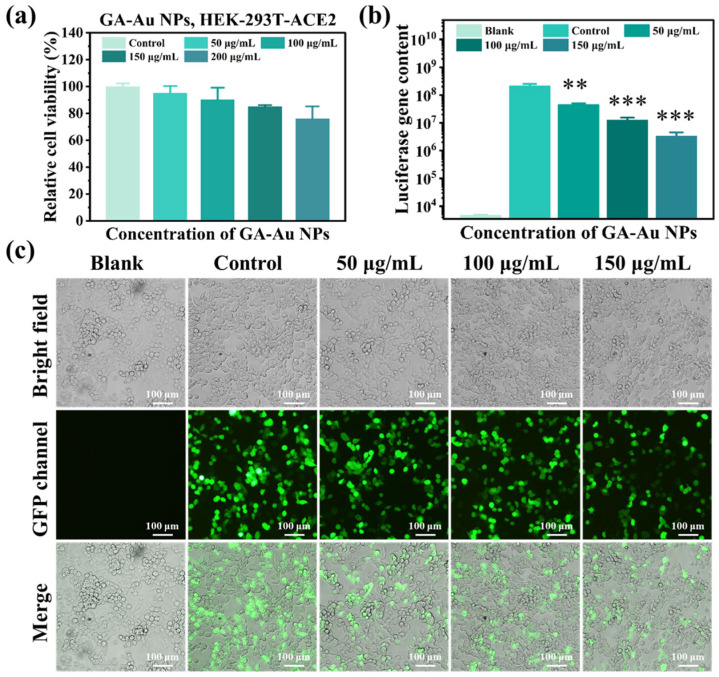
Cytocompatibility and inhibitory effect of GA-Au NPs on SARS-CoV-2 pseudovirus-associated infection in HEK-293T-ACE2 cells: (**a**) Cell viability of HEK-293T-ACE2 cells after exposure to GA-Au NPs (0–200 μg/mL) for 48 h. (**b**) Quantitative analysis of pseudovirus-associated infection based on luciferase activity in cell lysates measured 48 hpi, showing a concentration-dependent reduction following GA-Au NP pretreatment (0–150 μg/mL). *p* values were calculated using one-way ANOVA. ** *p* < 0.01, *** *p* < 0.001. (**c**) Representative bright-field, GFP, and merged fluorescence images showing decreased pseudovirus-associated GFP signals with increasing concentrations of GA-Au NPs. Scale bar = 100 μm.

Before antiviral activity was assessed, the biocompatibility of GA-Au NPs toward HEK-293T-ACE2 cells was examined to define an appropriate concentration range for the pseudovirus assay. HEK-293T-ACE2 cells are human cells engineered to express ACE2, the major entry receptor for SARS-CoV-2. As shown in Figure 8a, GA-Au NPs exhibited relatively low cytotoxicity after 48 h of incubation at concentrations up to 150 µg/mL, with cell viability remaining above approximately 80%. In contrast, at 200 µg/mL, cell viability decreased to below 80%, indicating a noticeable increase in cytotoxicity at this higher dose. Based on these results, 150 µg/mL was selected as the upper concentration limit for subsequent antiviral experiments to minimize the possibility that reductions in pseudovirus-associated signals were attributable to nonspecific cell death rather than nanoparticle-mediated antiviral effects.

We then quantified the effect of GA-Au NPs on pseudovirus infection using the firefly luciferase reporter signal, which reflects pseudovirus-associated infection efficiency in host cells under this assay system. HEK-293T-ACE2 cells were pretreated with different concentrations of GA-Au NPs (0–150 µg/mL) for 2 h and subsequently exposed to SARS-CoV-2 pseudovirus for 8 h; then, the cells were maintained in fresh medium for 48 h. As shown in Figure 8b, compared with the infected control group without GA-Au NP treatment, luciferase activity decreased progressively as the concentration of GA-Au NPs increased. Marked inhibition was observed in the 50, 100, and 150 µg/mL treatment groups, demonstrating a clear concentration-dependent reduction in pseudovirus-associated reporter activity. The statistically significant differences between the treated and control groups further support the reproducibility and reliability of this trend. Taken together, these data indicate that GA-Au NPs suppress SARS-CoV-2 pseudovirus-associated infection signals in vitro under the tested conditions.

The inhibitory effect observed in the luciferase assay was further supported by fluorescence microscopy. For each treatment group, bright-field, GFP, and merged images were collected to evaluate cell morphology and pseudovirus-associated GFP expression. The bright-field images showed that overall cell morphology and confluence were largely preserved across the tested concentration range, consistent with the cytotoxicity results shown in Figure 8a. In the GFP channel, strong green fluorescence was observed in the pseudovirus-infected control group, indicating efficient infection of HEK-293T-ACE2 cells. With increasing concentrations of GA-Au NPs, both the number of GFP-positive cells and the intensity of the fluorescent signal gradually declined. In particular, the 150 µg/mL group displayed a markedly reduced GFP signal compared with the untreated infected control. The merged images further illustrated the progressive decrease in pseudovirus-associated fluorescence with increasing nanoparticle concentration. These qualitative observations are consistent with the quantitative luciferase data and provide visual evidence that GA-Au NPs reduce SARS-CoV-2 pseudovirus-associated infection in a dose-dependent manner (Figure 8c).

## 4. Discussion

Glycyrrhizic acid-modified gold nanoparticles (GA-Au NPs) were successfully synthesized and characterized. Within the tested concentration range, the nanoparticles showed acceptable biocompatibility in both MARC-145 and HEK-293T-ACE2 cells, providing the basis for subsequent antiviral evaluation. Under these experimental conditions, GA-Au NPs inhibited PRRSV infection in vitro and also reduced reporter signals in a SARS-CoV-2 pseudovirus entry-related system, indicating antiviral activity in two enveloped virus-related models.

In the PRRSV system, GA-Au NPs reduced viral infectivity in a concentration-dependent manner, with an approximately 1.5-log decrease at the highest tested concentration. Although this level of inhibition does not indicate complete viral suppression, it demonstrates measurable antiviral activity in cell culture. In exploratory studies of antiviral nanomaterials, such activity remains meaningful when considered together with material stability, modifiable surface properties, and cellular compatibility [41]. The present results therefore support GA-Au NPs as a biologically active nanomaterial against PRRSV in vitro.

The stage-related assays further suggest that the antiviral effect of GA-Au NPs is not confined to a single step of the PRRSV infectious process. No significant inhibition was detected in the direct inactivation assay, indicating that GA-Au NPs did not show evident virucidal activity against free virions under the tested conditions. In contrast, inhibitory effects were observed in the adsorption, invasion, replication, and release assays. Moreover, the reduction in negative-strand RNA supports an effect on intracellular replication-associated events. Together, these findings indicate that GA-Au NPs interfere with multiple infection-related processes during PRRSV infection rather than acting primarily through direct extracellular virus inactivation.

This inhibition of release is consistent with several non-exclusive mechanisms. For an enveloped virus like PRRSV, egress often involves budding from cellular membranes. The GA-Au NPs, potentially through the membrane-active properties of the surface-conjugated glycyrrhizic acid, may interfere with the viral budding process, perhaps by integrating into lipid rafts or affecting membrane curvature. Alternatively, the nanoparticles could disrupt cellular secretory pathways or cytoskeletal networks essential for transporting and releasing viral particles [42,43]. The clear concentration dependence suggests that this interference requires a threshold occupancy of the nanoparticle at critical cellular sites.

The antiviral activity observed here may be related to the combined properties of the GA-Au NP construct. Glycyrrhizic acid has been reported to exhibit antiviral activity in several virus-related systems, including SARS-associated coronavirus and other enveloped viruses, and has also been linked to membrane-associated and immunomodulatory effects that may influence infection-related processes [44,45,46]. Gold nanoparticles, owing to their nanoscale dimensions, surface reactivity, and readily modifiable interfaces, have been widely investigated as antiviral materials or delivery platforms capable of affecting virus–cell interactions. In addition, surface functionalization can alter ligand presentation, interfacial binding behavior, and multivalent interactions, thereby influencing the biological performance of nanoparticle-based systems [21,47,48,49]. It is therefore plausible that GA modification contributes to the inhibitory effects observed in this study by affecting adsorption, cellular entry, and intracellular infection-associated events. However, the precise molecular basis of these effects remains to be clarified.

The inhibitory effect observed in the SARS-CoV-2 pseudovirus model further extends the antiviral profile of GA-Au NPs. In HEK-293T-ACE2 cells, GA-Au NPs decreased luciferase and GFP reporter signals in a concentration-dependent manner, consistent with inhibition in an entry-related pseudovirus assay. Pseudovirus systems are widely used to evaluate inhibitors of SARS-CoV-2-related entry and provide a practical platform for screening candidates that target early stages of infection [50,51]. At the same time, these systems do not fully reproduce the complete replication cycle of authentic viruses. Accordingly, the present findings should be interpreted as evidence of inhibitory activity in an entry-related surrogate model rather than as proof of efficacy against live SARS-CoV-2.

Several limitations of this study should be acknowledged. First, the antiviral assays were conducted in immortalized cell lines, and validation in more physiologically relevant systems, particularly primary porcine alveolar macrophages (PAMs) for PRRSV, would strengthen the biological relevance of the findings. Second, the relative contributions of free glycyrrhizic acid and unmodified Au NPs were not directly compared in parallel. Third, no benchmark antiviral compound was included for efficacy comparison. Future studies addressing these issues will be important for clarifying the contribution of each component, defining the underlying mechanism, and evaluating the translational potential of the GA-Au NP system.

## 5. Conclusions

GA-Au NPs are a biocompatible nanomaterial with detectable antiviral activity in vitro. In the PRRSV model, they reduced viral infectivity and interfered with multiple infection-related stages, including adsorption, invasion, replication, and release, without showing evident direct virucidal activity under the tested conditions. In a SARS-CoV-2 pseudovirus model, GA-Au NPs also reduced infection-associated reporter signals in an entry-related assay system. Overall, these findings support GA-Au NPs as a promising antiviral nanomaterial for further investigation in enveloped virus-related in vitro models.

## Data Availability

Data are contained within the article and the Supplementary Materials.

## Supplementary Materials

The following supporting information can be downloaded at: https://www.mdpi.com/article/10.3390/v18040454/s1, Table S1: Primer sequences for RT-qPCR detection of PRRSV; Table S2: Different synthetic schemes and corresponding particle sizes of GA-Au NPs; Figure S1: The UV-Vis absorption spectra of GA-Au NPs at different reaction conditions; Figure S2: The TEM images of GA-Au NPs at different reaction conditions; Figure S3: The UV-Vis absorption spectra of GA-Au NPs at 100 °C and different reaction times; Figure S4: The TEM images of GA-Au NPs at different reaction times.
