# Supplementary material for: Glycyrrhizic Acid-Modified Gold Nanoparticles Show Inhibitory Activity Against PRRSV and SARS-CoV-2 Pseudovirus In Vitro
Source: Viruses. 2026 Apr 9;18(4):454. doi: 10.3390/v18040454 (PMC13119537; doi:10.3390/v18040454)
Supplement: Supplementary file 1 [file viruses-18-00454-s001.zip › viruses-4200934-supplementary.pdf]

Supplementary materials:

Glycyrrhizic Acid-Modified Gold Nanoparticles Show Inhibitory Activity Against PRRSV and SARS-CoV-2 Pseudovirus In Vitro

Ting Tong [1,2,†,*], Xiaotong Zhang [2,†], Yating Lei [1], Linjie Li [1], Shaobo Xiao [3], and Jiangong Liang [2,3,*]

1. Materials and Methods

1.1. Chemicals and Reagents

Tetrachloroauric(III) acid trihydrate ($HAuCl_4 \cdot 3H_2O$, AR), sodium hydroxide (NaOH, AR) from Sinopharm Chemical Reagent Co., Ltd.; Glycyrrhizic acid ($C_{42}H_{62}O_{16}$, ≥93%) was obtained from Shanghai Aladdin Chemical Reagent Co., Ltd.; Unless otherwise specified, all aqueous solutions were prepared using Milli-Q deionized water (Millipore, 18.2 MΩ·cm). All chemicals and reagents were used as received without further purification.

1.2. Characterization of GA-Au NPs

The physicochemical properties of the synthesized GA-Au NPs were comprehensively characterized using a suite of analytical techniques. The morphology and lattice structure were examined by high-resolution transmission electron microscopy (HRTEM) on a JEM-2100F instrument (JEOL, Japan). Crystalline structure was determined by powder X-ray diffraction (XRD) on a Bruker D8 Advance diffractometer (Germany) using Cu Kα radiation (λ = 1.5406 Å) operated at 40 kV and 40 mA, with a scanning range of 30° to 90° (2θ). The surface plasmon resonance property was assessed by ultraviolet-visible (UV-Vis) spectroscopy on a Shimadzu UV-2450 spectrophotometer (Japan). Hydrodynamic diameter (size distribution) was measured in triplicate using dynamic light scattering (DLS) on a Malvern Zetasizer Nano ZS instrument (UK). Surface chemical composition and elemental states were analyzed by X-ray photoelectron spectroscopy (XPS) on an ESCALAB Xi^+ spectrometer (Thermo Fisher Scientific, USA) with a monochromatic Al Kα X-ray source. Chemical functional groups and the successful conjugation of GA were verified by Fourier transform infrared (FT-IR) spectroscopy on a Nicolet Avatar-330 spectrometer (Thermo Fisher Scientific, USA) in the range of 4000-500 cm^{-1}. Confocal fluorescence imaging for the antiviral assays was performed using an Olympus FV10 laser scanning confocal microscope (Japan). The fluorescence intensity of green fluorescent protein (GFP) encoded by SARS-CoV-2 pseudovirus infection was detected by Ti-U inverted fluorescence microscope from Nikon Corporation in Japan.

1.3. Indirect Immunofluorescence Assay (IFA)

Briefly, cells were fixed with 4% paraformaldehyde (15 min, RT) and permeabilized with pre-chilled methanol (10 min, -20 °C). After PBS washes, non-specific binding was blocked with 5% BSA (w/v) for 45 min. Cells were then incubated with a primary antibody against the PRRSV N protein

(mouse monoclonal), followed by an Alexa Fluor 594-conjugated donkey anti-mouse IgG secondary antibody. Nuclei were stained with DAPI. Following final washes, images were captured using an Olympus FV10 laser scanning confocal microscope.

1.4. Plaque Assay

The viral titer was quantified by standard plaque assay. After incubating MARC-145 cells in 6-well plates with the PRRSV, the inoculum was removed, and cells were washed three times with PBS to eliminate unbound virus. An overlay medium, consisting of 2× DMEM, 1.8% low-melting-point agarose, FBS, and penicillin-streptomycin (48:48:3:1 ratio), was added to each well. Plates were chilled at 4°C for 15 min to solidify the overlay and then incubated at 37°C for 2–3 days. Plaques were subsequently stained with 1 mL of neutral red solution (0.33 mg/mL) for 1 h, counted, and the viral titer was calculated and expressed as plaque-forming units per mL (PFU/mL).

1.5. RNA Extraction and RT-qPCR Analysis

To quantitatively assess the effect of GA-Au NPs on PRRSV replication, the level of viral RNA was measured by reverse transcription quantitative polymerase chain reaction (RT-qPCR).

Total RNA Extraction:

Total RNA was isolated from virus-infected cells using RNA Solv® Reagent (Omega Bio-tek) following the manufacturer's protocol, with all steps performed using RNase-free materials. Briefly, cell pellets (~10^7 cells) were lysed in 1 mL of reagent and incubated for 10 min at room temperature. Subsequently, 200 μL of chloroform was added, and the mixture was vortexed vigorously for 30 s followed by incubation on ice for 10 min. After centrifugation (12,000 × g, 10 min, 4°C), the upper aqueous phase (~500 μL) was transferred to a new tube and mixed with an equal volume of isopropanol to precipitate the RNA. Following a 10 min incubation at room temperature and centrifugation (12,000 × g, 10 min, 4°C), the RNA pellet was washed once with 75% ethanol, air-dried, and dissolved in DEPC-treated water. RNA concentration and purity (A260/A280 and A260/A230 ratios) were measured using a Nanodrop spectrophotometer. Qualified RNA samples were diluted to 500 ng/μL for subsequent use.

cDNA Synthesis:

cDNA was synthesized from the extracted RNA using the Transcriptor First Strand cDNA Synthesis Kit (Roche). For the synthesis of cDNA from cellular genes and the positive-strand viral RNA, 1 μg of total RNA was mixed with 1 μL of Oligo(dT)18 primer and RNase-free water to a total volume of 13 μL. The mixture was incubated at 65 °C for 10 minutes and then immediately placed on ice.

To specifically detect the negative-strand RNA of PRRSV (a key replication intermediate), a strand-specific primer was used. In this case, 1 μg of total RNA was mixed with 1 μL of the PRRSV negative-strand specific primer (5'-UF: GACGTATAGGTGTTGGCTC-3') and RNase-free water to a total volume of 13 μL, followed by the same incubation conditions.

Subsequently, 7 μL of a master mix containing 2 μL of deoxynucleotide mix, 4 μL of 5× Reverse Transcription Buffer, 0.5 μL of RNase Inhibitor, and 0.5 μL of Reverse Transcriptase was added to the 13 μL primer-RNA mixture, making a final reaction volume of 20 μL. The reverse transcription

reaction was carried out at 55 °C for 30 minutes, followed by enzyme inactivation at 85 °C for 5 minutes. The synthesized cDNA used directly for qPCR.

Quantitative Real-Time PCR (qPCR):

The abundance of PRRSV RNA was quantified by qPCR targeting a region of the 5'UTR region. The sequences of the specific primers used are listed in **Table 1**.

Table S1. Primer sequences for RT-qPCR detection of PRRSV.

Primer Name	Sequence (5' → 3')
5'UTR-F	GCATTTGTATTGTCAGGAGC
5'UTR-R	AGCAGTGCAACTCCGGAAG

A standard curve for absolute quantification was generated using a plasmid containing the 5'UTR target sequence with known copy numbers (serial dilutions from 1×10^{10} to 1×10^{4} copies/μL). For quantitative analysis, real-time PCR was set up in 10 μL reactions containing the following components: 5.0 μL of 2× LightCycler® 480 SYBR Green I Master mix, 0.4 μL each of forward and reverse primers (at a concentration of 10 μmol/L), 1.0 μL of cDNA, and RNase-free water to make up the final volume. The thermal profile, run on an ABI ViiA 7 instrument, commenced with a 10-min denaturation at 95 °C, succeeded by 40 cycles of denaturation at 95 °C for 15 s, primer annealing at 56 °C for 15 s, and extension at 72 °C for 30 s. Fluorescence reading was taken at the completion of each extension step. Quantification of viral RNA copy numbers was achieved by referencing the generated standard curve.

2. Results

Table S2. Different synthetic schemes and corresponding particle sizes of GA-Au NPs.

Number	Heating temperature ($HAuCl_4$ solution)	+ GA, reaction time after discoloration	Reaction conditions after discoloration	Product color	Hydrated particle size
**a**	100 °C	20 min	25 °C (90 min)	Blood red	18.9 nm
**b**	100 °C	0 min	25 °C (90 min)	Blue purple	25.8 nm
**c**	90 °C	20 min	25 °C (90 min)	Purplish red	25.4 nm
**d**	70 °C	20 min	25 °C (90 min)	lilac colour	32.8 nm

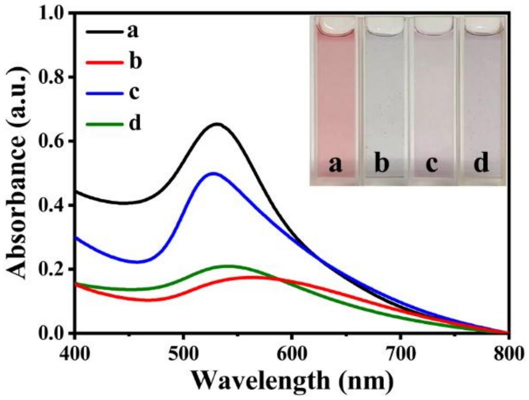

Figure S1. The UV-Vis absorption spectra of GA-Au NPs at different reaction conditions. The reaction conditions corresponding to a, b, c and d are shown in **Table 1**.

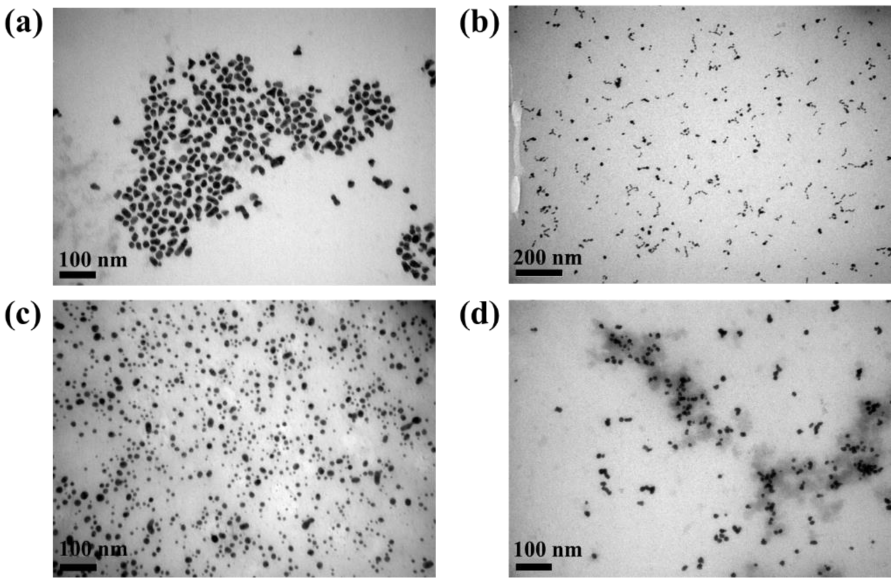

Figure S2. The TEM images of GA-Au NPs at different reaction conditions. The reaction conditions corresponding to a, b, c and d are shown in **Table 1**.

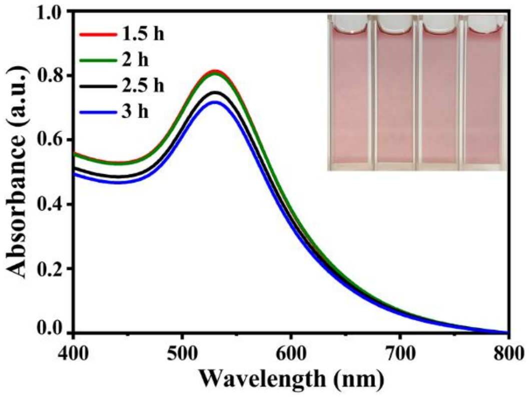

Figure S3. The UV-Vis absorption spectra of GA-Au NPs at 100 °C and different reaction times (from left to right represent GA-Au NPs synthesized with reaction times of 1.5 h, 2 h, 2.5 h and 3 h).

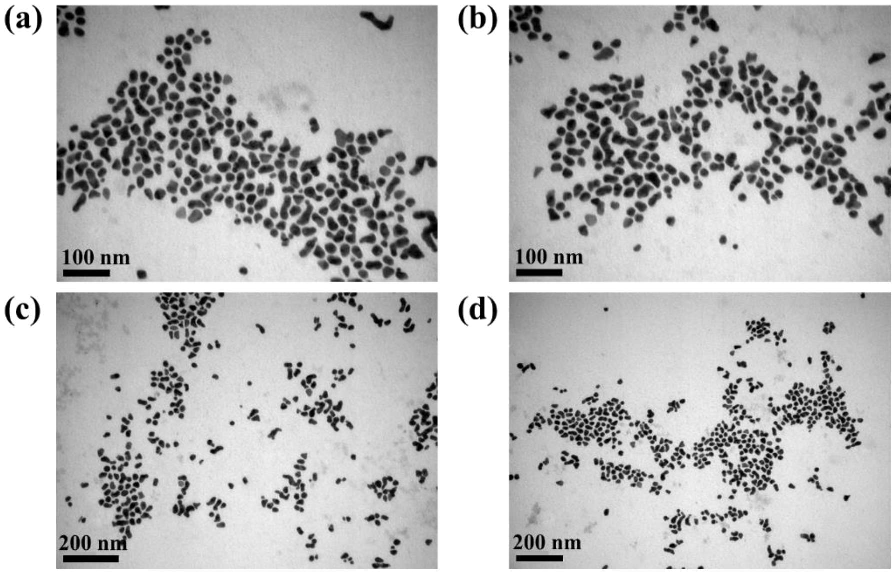

Figure S4. The TEM images of GA-Au NPs at 100 °C for 1.5 h (a), 2 h (b), 2.5 h (c) and 3 h (d).
